# Regulation of Leukocyte Recruitment to the Spleen and Peritoneal Cavity during Pristane-Induced Inflammation

**DOI:** 10.1155/2017/9891348

**Published:** 2017-10-19

**Authors:** Yu Li, Junping Wu, Long Xu, Qi Wu, Zhen Wan, Li Li, Hongzhi Yu, Xue Li, Kuan Li, Qiuyang Zhang, Zhili Hou, Xin Sun, Huaiyong Chen

**Affiliations:** ^1^Department of Basic Medicine, Haihe Clinical College of Tianjin Medical University, Tianjin, China; ^2^Key Research Laboratory for Infectious Disease Prevention for State Administration of Traditional Chinese Medicine, Tianjin Institute of Respiratory Diseases, Tianjin Haihe Hospital, Tianjin 300350, China; ^3^Department of Respiratory Medicine, Tianjin Haihe Hospital, Tianjin 300350, China; ^4^Department of Tuberculosis, Tianjin Haihe Hospital, Tianjin 300350, China

## Abstract

Chronic inflammation is associated with an increased number of leukocytes in the spleen, which are then redirected to the site of inflammation. However, it remains unknown how leukocyte recruitment is regulated. Herein, chronic inflammation was induced by intraperitoneal injection of pristane into mice. Leukocytes in the spleen or in the peritoneal cavity were quantified by flow cytometry. We found that the loss of IL-6 decreased macrophage recruitment to the spleen and the peritoneal cavity during pristane-induced inflammation. The loss of TNF*α* delayed the recruitment of neutrophils and macrophages to the spleen and inhibited the recruitment of neutrophils, macrophages, B cells, and T cells. The recruitment of neutrophils and macrophages into the spleen or peritoneal cavity was largely inhibited in the absence of LT*α*. The loss of TNF*α* receptor 1/2 resulted in reduced recruitment of neutrophils, macrophages, and dendritic cells into the spleen, but only neutrophil recruitment was inhibited in the peritoneal cavity. Similarly, a lack of B cells significantly impeded the recruitment of neutrophils, macrophages, and dendritic cells to the spleen. However, only macrophage recruitment was inhibited in the absence of T cells in the spleen. These data provide insight into the development of chronic inflammation induced by noninfectious substances.

## 1. Introduction

Noninfectious inflammation can be induced by persistent indigestible substances. In many studies, a hydrocarbon oil pristane is often injected intraperitoneally to model noninfectious inflammation. Pristane administration induces macrophage activation [1, 2]. Depending on the genetic background of the model, pristane injection can trigger a local inflammatory response (lipogranuloma), erosive arthritis that resembles rheumatoid arthritis, and systemic lupus erythematosus, followed by autoantibody formation and many clinical manifestations [3–5]. Previous studies from our group and other labs have demonstrated that oil granulomas represent the major pathology in response to pristane injections in C57BL/6 mice [2, 3]. Pristane-induced chronic inflammation has been characterized by the continuous recruitment of leukocytes, including lymphocytes, neutrophils, and macrophages, to the peritoneal cavity and the spleen [6–8].

We and others have discovered factors that control the recruitment of inflammatory leukocytes to the peritoneal mesentery in response to pristane [9, 10]. Cytokines are known to regulate the migration of neutrophils and macrophages during inflammation. Tumor necrosis factor alpha (TNF*α*) stimulates the expression of monocyte chemoattractant protein 1 (MCP-1) [11]. Mice deficient for TNF*α* develop defective oil granulomas with reduced recruitment of macrophages and neutrophils [10]. Interleukin-6 (IL-6) seems to regulate both plasmacytoma development in BALB/c mice and oil granuloma formation in C57BL/6 mice during pristane-induced inflammation [10, 12]. Lymphotoxin alpha (LT*α*) and TNF*α* were shown to induce the expression of homing chemokines in B and T cell areas of the spleen [13]. LT*α* is also required for the recruitment of dendritic cells, neutrophils, and macrophages to the mesentery in response to pristane [10]. Beyond that, LT*α* also maintains the structure of the mature marginal sinus (MS) in the postnatal spleen [14]. In addition to lymphocytes, dendritic cells can also produce LT*α* [15]. TNF*α* plays an important role in the formation of primary B cell follicles and follicular dendritic cells [16]. TNF*α* is mainly secreted by primitive neutrophils and participates in the inflammatory response involved in rheumatoid arthritis and inflammatory bowel disease [17].

As the two major cell types in the spleen, B cells and T cells produce cytokines and chemokines [18]. The migration of inflammatory leukocytes, including dendritic cells, neutrophils, and macrophages, to the peritoneal mesentery has been shown to be promoted in LAT^−/−^ (lack mature T cells) mice but inhibited in *μ*MT (lack mature B cells) mice during the pristane-induced immune response [10]. Thus, leukocyte migration to inflammatory sites in response to pristane has been well characterized. Our previous data demonstrated that lymphocytes and their produced cytokines including TNF*α*, LT*α*, and IL-6 play an important role in such process. We hypothesized that these factors may also be critical for the recruitment of leukocytes to the spleen during pristane-induced inflammation.

In the present study, chronic inflammation was induced in C57BL/6J mice by intraperitoneal injection of pristane to determine whether or not B cells, T cells, IL-6, TNF*α*, and LT*α* were involved in pristane-induced inflammation via the regulation of dendritic cell, neutrophil, and macrophage recruitment to the spleen. Using flow cytometry to quantitatively analyze the number of leukocytes in the spleen, we observed that the recruitment of dendritic cells, neutrophils, and macrophage to the spleen followed different regulatory patterns.

## 2. Materials and Methods

### 2.1. Mouse Strains and Pristane Administration

*μMT* mice [19], *TNFα*^−/−^ mice, *LTα*^−/−^ mice, *IL-6*^−/−^ mice, *TNFR1/2*^−/−^ mice, and *LAT*^−/−^ mice were purchased from the Jackson Laboratories (Bar Harbor, ME, USA). In these studies, all animals were housed and cared for in a specific pathogen-free facility with sterile bedding, water, and food. Therefore, any effects of incidental antigen exposure on inflammation were minimized. All animal experiments were approved by the Animal Care and Use Committee at Haihe Clinical College of Tianjin Medical University. To induce granuloma inflammation, adult mice (8–10 weeks old) received a single intraperitoneal (i.p.) injection of pristane (100 or 300 *μ*L; 2.8 × 10^−4^ or 8.3 × 10^−4^ mol, resp., ≥95% purity, Sigma-Aldrich, St. Louis, MO, USA). Age-matched, untreated mice were used as controls. Three to six WT mice or knockout mice were sacrificed at each time point after pristane.

### 2.2. Preparation of Splenic Cells and Resident Peritoneal Cells

After anesthesia with i.p. injection of pentobarbital sodium (100 mg/kg), cervical dislocation was then performed to sacrifice mice at various time points following an injection of pristane. The abdominal cavity was opened, and the complete spleen was removed from C57BL/6J mice or the specific gene knockout mice. Spleens were minced in RPMI-1640 media (Gibco) at room temperature (21°C) to prepare single-cell suspensions. The resulting cell suspension was removed and filtered through a fine nylon mesh (Denville Scientific Inc., Metuchen, NJ, USA). Resident peritoneal cavity (PC) cells were collected by lavage with 10 mL of ice-cold RPMI-1640 supplemented with 5% FBS. After centrifugation, the cell pellets were harvested and counted with a cytometer.

### 2.3. Flow Cytometry

Suspensions of splenic cells or peritoneal cells prepared above were incubated with ammonium chloride buffer for 1 min on ice to lyse red blood cells (RBCs) before immunolabeling. Nucleated cells were labeled with antibodies specific for B220 (PE-Cy7), TCR*β* (APC), CD11c (PE), Gr-1 (FITC), and CD11b (APC-Cy7) for 20 min on ice. To identify and exclude dead cells, DAPI (7-AAD Viability Staining Solution, eBioscience) was used. Flow cytometric data were analyzed with FlowJo software (Tree Star, Ashland, OR, USA). Labeled cells were analyzed in a FACSVantage with DIVA option. The absolute number of each cell type in each sample was determined by multiplying the total number of cells with the percentage of each cell type in the same sample.

### 2.4. Quantitative PCR

Mice were anesthetized and sacrificed as mentioned above. The peritoneal mesentery was harvested and minced. Total RNA was extracted from the tissue using TRIzol reagent (Invitrogen, Carlsbad, CA, USA). Messenger RNA was reverse transcribed with oligo (dT) primer for 1 h at 50°C. Quantitative PCR was performed in an iCycler Thermal Cycler with SYBR® Green PCR core reagents (Applied Biosystems, Foster City, CA) and primers for specific genes. Amplification conditions were as follows: denaturation at 94°C for 10 min and amplification at 94°C for 15 s and 60°C for 45 s, repeated for 40 cycles. Primers included were as follows: *β*-actin, forward 5′-AGCCATGTACGTAGCCATCC-3 and reverse 5′-CTCTCAGC TGTGGTGGTGAA-3′; TNF*α*, forward 5′-TCCCCAAAGGGATGAGAAGTTC-3′ and reverse 5′-GGGAGTAGACAAGGTACAAC-3′; IL-1*β*, forward 5′-GGTACATCAGCACCTCACAA-3′ and reverse 5′-TTAGAAACAGTCCAGCCCATAC-3′; and IL-6, forward 5′-CCCAACAGACCTGTCTATACC-3′ and reverse 5′-CAGCTTATCTGTTAGGAGAGC-3′. The relative mRNA levels for the indicated target genes were calculated by the comparative Ct (threshold cycle) method and normalized to *β*-actin levels in the same sample.

### 2.5. Statistical Analyses

Data were collected from three or more replicate samples in each independent experiment. Differences between paired groups were analyzed using Student's *t*-test; *p* values ≤ 0.05 were considered significant.

## 3. Results

### 3.1. Dose-Dependent Recruitment of Leukocytes to the Spleen

**Figure 1:**
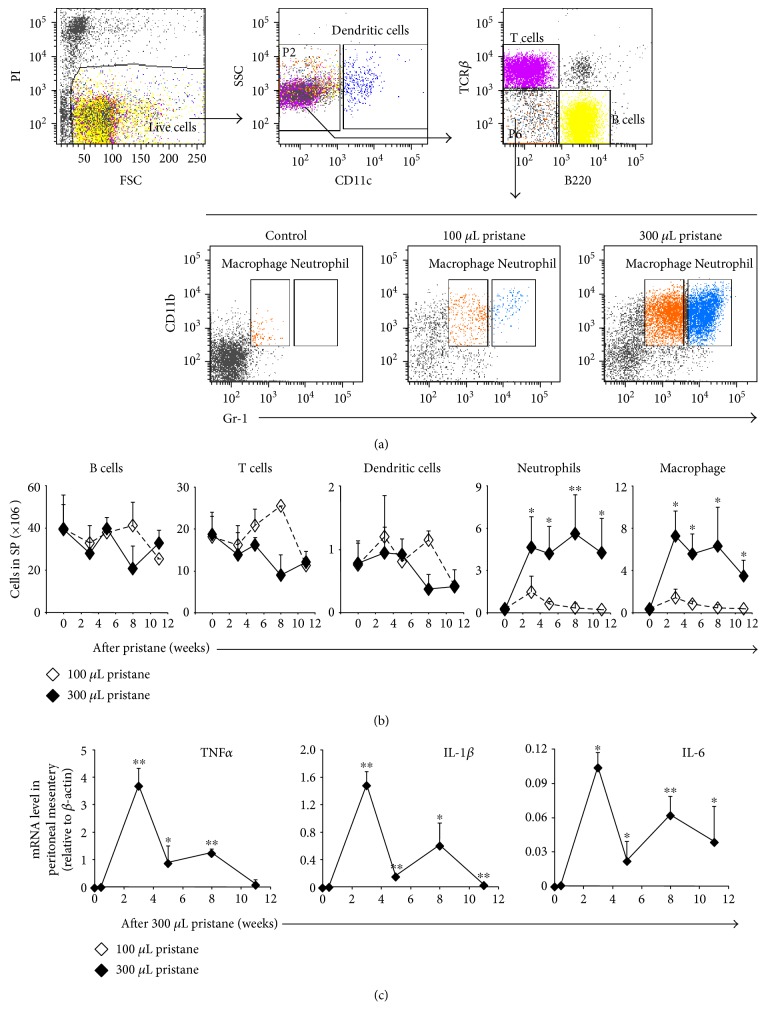
Leukocyte responses to pristane treatment were dose dependent. Whole splenic cells were analyzed using flow cytometry after staining with antibodies specific for B220, TCR*β*, CD11c, CD11b, and Gr-1 (a). The number of B cells (B220^+^), T cells (TCR*β*^+^), dendritic cells (CD11c^+^), macrophages (CD11b^+^Gr1^low^), or neutrophils (CD11b^+^/Gr1^hi^) in the spleen was quantified (b). The difference between 100 *μ*L of pristane treatment and 300 *μ*L of pristane treatment in C57BL/6J mice is shown. (c) Peritoneal mesentery was collected from C57BL/6 mice treated with 300 *μ*L of pristane and analyzed for the expression of cytokines. The difference between 300 *μ*L pristane-treated and nontreated C57BL/6J mice is shown. Data represent two independent experiments. ^∗^*p* < 0.05 and ^∗∗^*p* < 0.01.

To investigate splenic leukocyte responses to pristane, C57BL/6J mice were injected intraperitoneally with a single dose of 100 or 300 *μ*L of pristane. Mice were then sacrificed to prepare single-cell suspensions at various time points. Whole splenic cells were analyzed using flow cytometry after staining with antibodies specific for B220, TCR*β*, CD11c, CD11b, and Gr-1. CD11c^+^ dendritic cells, CD11c^−^/B220^+^ B cells, CD11c^−^/TCR*β*^+^ T cells, CD11c^−^/CD11b^+^/Gr-1^hi^ neutrophils, and CD11c^−^/CD11b^+^/Gr-1^low^ macrophages were fractionated and counted (Figure 1(a)). The number of B cells, T cells, and dendritic cells in the spleen was unchanged during pristane treatment at both doses (Figure 1(b)). However, the responses of neutrophils and macrophages to pristane were dose dependent (Figure 1(b)). In the presence of low-dose pristane, the number of both neutrophils and macrophages was augmented at week three, which then decreased and returned to their basal levels at 11 weeks posttreatment (Figure 1(b)). In contrast, the injection of high-dose pristane resulted in the recruitment of significantly more neutrophils and macrophages to the spleen throughout the treatment period (Figure 1(b)). Therefore, 300 *μ*L of pristane was chosen for the subsequent experiments.

We also collected mesenteric tissues from the peritoneal cavity and observed that the mRNA of the inflammatory cytokine TNF*α* was rapidly elevated at week three after treatment with 300 *μ*L of pristane and then gradually returned to the basal level by week 11 (Figure 1(c)). Likewise, the expression of IL-1*β* and IL-6 in the peritoneal mesentery followed a similar trend during pristane-induced inflammation (Figure 1(c)). These data suggested that TNF*α* and IL-6 are associated with pristane-induced inflammation and may play a role in the recruitment of leukocytes to the spleen.

### 3.2. IL-6 Promotes Recruitment of Macrophages to the Spleen in Response to Pristane

**Figure 2:**
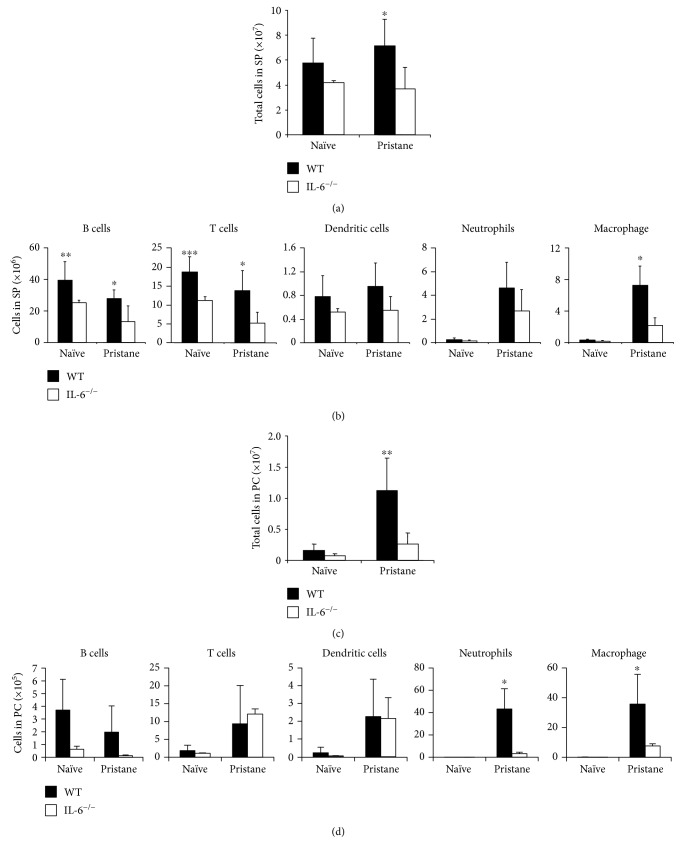
IL-6 deficiency decreased the recruitment of splenic macrophages in response to pristane treatment. (a, b) *IL-6*^−/−^ mice and WT mice were injected with 300 *μ*L (8.3 × 10^−4^ mol) of pristane and sacrificed at three weeks thereafter. Single cell suspensions of the spleen (SP) and peritoneal cavity (PC) from pristane-injected WT and *IL-6*^−/−^ mice were prepared for cellularity analysis by flow cytometry. The total cell number and the number of each cell type were calculated, respectively. (c, d) The total number of cells or the number of each cell type in the PC was analyzed in the same way. The difference between WT and *IL-6*^−/−^ mice is shown. Data represent two independent experiments. ^∗^*p* < 0.05, ^∗∗^*p* < 0.01, and ^∗∗∗^*p* < 0.001.

To investigate a possible role IL-6 in leukocyte recruitment to the spleen during pristane-induced inflammation, we injected IL-6^−/−^ mice with 300 *μ*L of pristane intraperitoneally. At the steady state, the total number of splenic cells decreased slightly in IL-6^−/−^ mice as compared to the total number of splenic cells in wild-type (WT) mice (Figure 2(a)), which may be due to the significant reduction in the number of B cells and T cells (Figure 2(b)). The number of B cells and T cells remained attenuated in IL-6^−/−^ mice when compared to the number of B cells and T cells in WT mice three weeks after pristane treatment (Figure 2(b)). Pristane treatment promoted the recruitment of both neutrophils and macrophages to the spleen (Figure 2(b)). In the absence of IL-6, the recruitment of neutrophils in response to pristane treatment remained intact (Figure 2(b)). However, the recruitment of macrophages was inhibited in the absence of IL-6 during pristane-induced inflammation (Figure 2(b)). In the peritoneal cavity, pristane injection led to an increased infiltration of cells, which was abolished by IL-6 loss (Figure 2(c)). Neutrophils and macrophages represented the two major mononuclear cell infiltrates in the peritoneal cavity after pristane treatment (Figure 2(d)). IL-6 loss significantly inhibited the infiltration of neutrophils and macrophages into the peritoneal cavity after pristane treatment (Figure 2(d)). These data demonstrated that IL-6 is required for the infiltration of both neutrophils and macrophages into the peritoneal cavity and for the recruitment of only macrophages to the spleen during pristane-induced inflammation.

### 3.3. LT*α* Enhanced the Recruitment of Both Neutrophils and Macrophages to the Spleen in Response to Pristane

**Figure 3:**
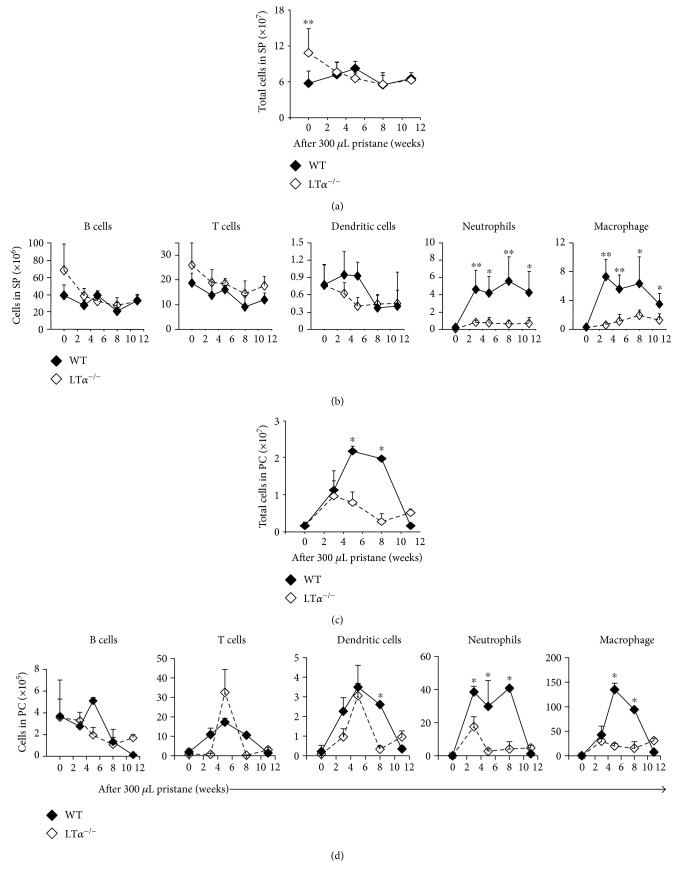
LT*α* loss inhibited the recruitment of both splenic neutrophils and macrophages in response to pristane treatment. (a, b) *LT*α^−/−^ mice and WT mice were injected with 300 *μ*L (8.3 × 10^−4^ mol) of pristane and were then sacrificed at the indicated time points. The total number of cells and the number of each cell type were calculated. (c, d) The total number of cells or the number of each cell type in the PC was also analyzed. The difference between WT and *LTα*^−/−^ mice is shown. Data represent two independent experiments. ^∗^*p* < 0.05 and ^∗∗^*p* < 0.01.

To determine whether LT*α* affects recruitment of leukocytes to the spleen, *LTα*^−/−^ mice were injected with 300 *μ*L of pristane. At the steady state, the total number of splenic cells in *LTα*^−/−^ mice was higher than the total number of splenic cells in WT mice (Figure 3(a)), which may be due to the slight increase in B cells and T cells (Figure 3(b)). LT*α* deficiency had little effect on the number of dendritic cells at the steady state. During pristane-induced inflammation, the recruitment of both neutrophils and macrophages to the spleen was significantly blocked (Figure 3(b)). Likewise, the infiltration of neutrophils and macrophages into the peritoneal cavity was also inhibited in the absence of LT*α*, resulting in a decrease in the number of total cell infiltrates during pristane-induced inflammation (Figures 3(c) and 3(d)). It appeared that the dendritic cells were cleared faster from the peritoneal cavity in the LT*α*^−/−^ mice than in the WT mice (Figure 3(d)). This observation suggested that LT*α* is critical for the recruitment of both neutrophils and macrophage in the spleen and the peritoneal cavity during pristane-induced inflammation.

### 3.4. TNF*α* Is Required for Rapid Mounting of the Inflammatory Response to Pristane in the Spleen

**Figure 4:**
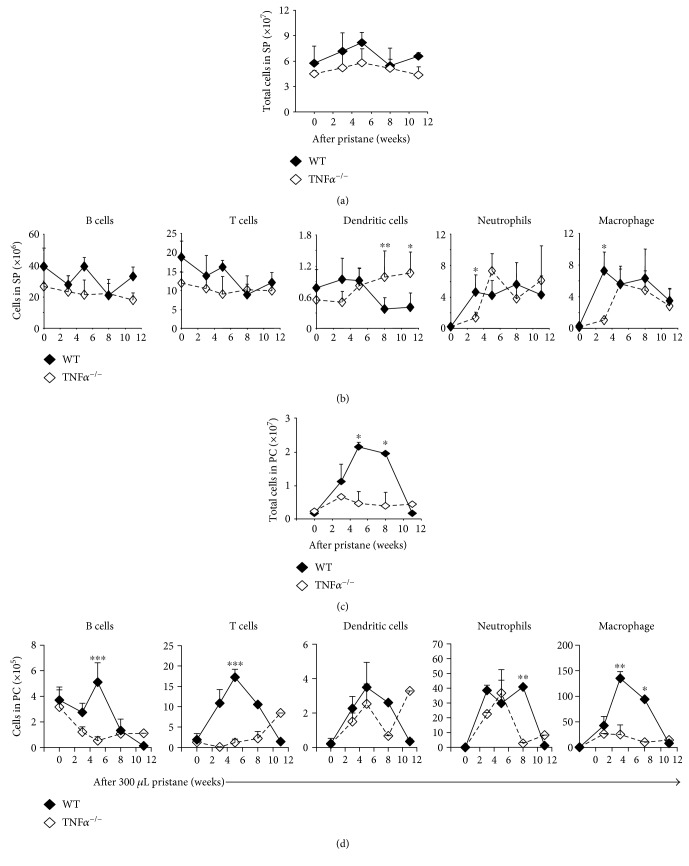
Lack of TNF*α* delayed the recruitment of splenic neutrophils and macrophages in response to pristane treatment. (a, b) *TNFα*^−/−^ mice and WT mice were injected with 300 *μ*L (8.3 × 10^−4^ mol) of pristane and were then sacrificed at the indicated time points. The total number of cells and the number of each cell type were calculated. (c, d) The total number of cells or the number of each cell type in the PC was also analyzed. The difference between WT and *TNFα*^−/−^ mice is shown. Data represent two independent experiments. ^∗^*p* < 0.05, ^∗∗^*p* < 0.01, and ^∗∗∗^*p* < 0.001.

TNF*α*^−/−^ mice were also used in the present study to examine whether or not TNF*α* regulates the inflammatory response to pristane in the spleen. For this, *TNFα*^−/−^ mouse and WT mouse controls were injected intraperitoneally with 300 *μ*L of pristane. TNF*α* loss had little to no effect on the total number of splenic cells at the steady state or during pristane-induced inflammation (Figure 4(a)). However, dendritic cells seemed to increase in the absence of TNF*α* and after pristane treatment (Figure 4(b)). The number of both neutrophils and macrophages in the spleen was significantly lower in TNF*α*^−/−^ mice than the number of neutrophils and macrophages in WT mice at week three but was similar at week five after pristane treatment (Figure 4(b)). In the peritoneal cavity, cell infiltration was largely inhibited (Figure 4(c)). Among all cell types, fewer B cells and T cells infiltrated into the peritoneal cavity after pristane treatment (Figure 4(d)). Clearance of neutrophils was faster in the absence of TNF*α*, while macrophage recruitment was almost completely blocked (Figure 4(d)). These data suggested that TNF*α* is required for the rapid response to pristane in the spleen but exhibited a more complicated regulatory role in the immune response to pristane in the peritoneal cavity.

### 3.5. TNFR1/2 Promoted Recruitment of Neutrophils and Macrophages to the Spleen

**Figure 5:**
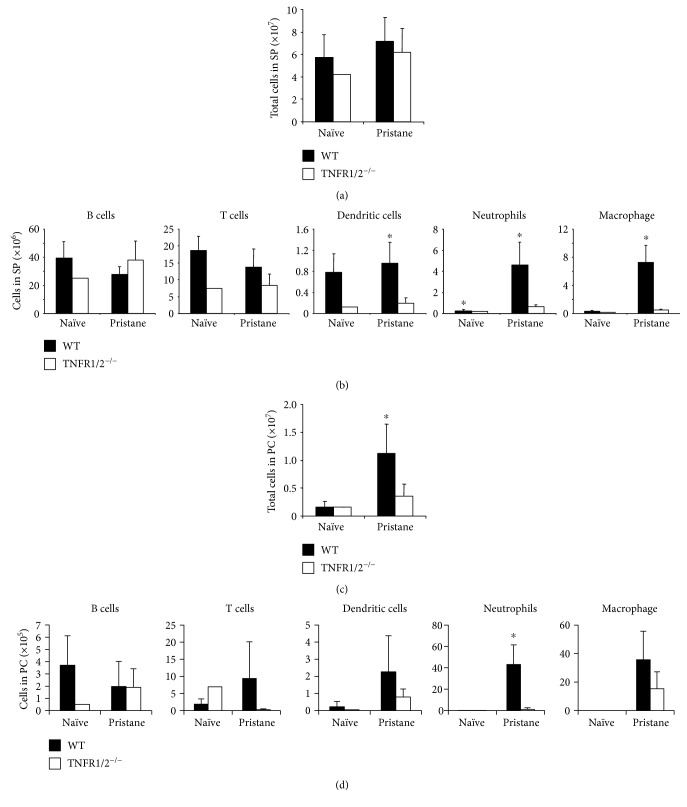
The absence of TNFR1/2 inhibited the recruitment of splenic neutrophils, macrophages, and dendritic cells in response to pristane treatment. (a, b) *TNFR1/2*^−/−^ mice and WT mice were injected with 300 *μ*L (8.3 × 10^−4^ mol) of pristane and sacrificed at three weeks thereafter. Total number of cells and the number of each cell type were calculated. (c, d) Total number of cells and the number of each cell type in the PC were also analyzed. The difference between WT and *TNFR1/2*^−/−^ mice is shown. Data represent two independent experiments. ^∗^*p* < 0.05.

As TNF*α* was shown to regulate pristane-induced inflammation, we predicted that TNF*α* receptors may also play a role. Using TNFR1/2^−/−^ mice to examine this hypothesis, the recruitment of both neutrophils and macrophages to the spleen was largely inhibited in the absence of TNFR1/2, as expected; furthermore, the total number of splenic cells remained unchanged three weeks after pristane treatment (Figures 5(a) and 5(b)). Moreover, the dendritic cells in the spleen also appeared to have their recruitment inhibited (Figure 5(b)). In the peritoneal cavity, TNFR1/2 loss resulted in an attenuation of cell infiltration after pristane treatment (Figure 5(c)). Macrophage recruitment to the peritoneal cavity remained intact in response to pristane treatment even in the absence of TNFR1/2, but neutrophil recruitment was almost fully inhibited (Figure 5(d)). These data suggested that TNF*α* receptors play a more profound role than TNF*α* in regulating pristane-induced inflammation.

### 3.6. B Cells Promote the Recruitment of Neutrophils and Macrophages to the Spleen

**Figure 6:**
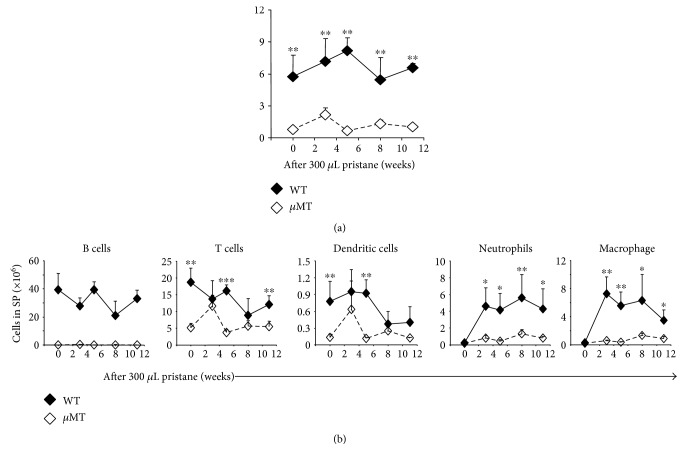
B cell loss reduced the cellularity of both leukocytes and lymphocytes during pristane-induced splenic inflammation. (a, b) *μMT* mice and WT mice were injected with 300 *μ*L (8.3 × 10^−4^ mol) of pristane and were then sacrificed at the indicated time points. The total number of cells and the number of each cell type were calculated. The difference between WT and *μMT* mice is shown. Data represent two independent experiments. ^∗^*p* < 0.05, ^∗∗^*p* < 0.01, and ^∗∗∗^*p* < 0.001.

As a major cell type in the spleen, B cells are known to produce inflammatory cytokines, including TNF*α*. Next, we sought to examine whether or not B cells were involved in the pristane-induced inflammatory response by utilizing *μMT* mice, which lack B cells. At the steady state, loss of B cells led to a significant drop in the number of T cells and dendritic cells, as well as the total number of splenic cells (Figures 6(a) and 6(b)). Following pristane-induced inflammation, the number of all cell types analyzed, including T cells, dendritic cells, neutrophils, and macrophages, was reduced in the absence of B cells when compared to the number of cell of each type in the WT controls (Figure 6(b)). This finding suggested that B cells are crucial for the recruitment of dendritic cells, neutrophils, and macrophages during the pristane-induced inflammatory response in the spleen.

### 3.7. T Cells Were Crucial for the Recruitment of Splenic Macrophages in Response to Pristane

**Figure 7:**
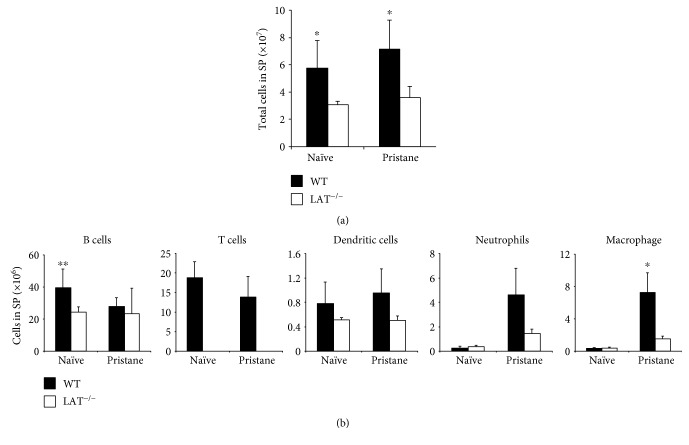
T cell loss inhibits splenic macrophage recruitment during pristane-induced splenic inflammation. (a, b) *LAT^−/−^* mice and WT mice were injected with 300 *μ*L (8.3 × 10^−4^ mol) of pristane and sacrificed at three weeks thereafter. Total number of cells and the number of each cell type were calculated. The difference between WT and *LAT^−/−^* mice is shown. Data represent two independent experiments. ^∗^*p* < 0.05 and ^∗∗^*p* < 0.01.

We next sought to examine whether or not T cells, the other major cell type in the spleen, were important in the pristane-induced inflammatory response. To do so, we utilized *LAT*^−/−^ mice. At the steady state, loss of T cells led to a significant reduction in the number of B cells, as well as the resulting reduction in total number of splenic cells (Figures 7(a) and 7(b)). Three weeks postpristane injection, only the number of macrophages was reduced in the absence of T cells, as compared to T cell abundance in the WT controls (Figure 7(b)). This finding suggested that T cells are important for the recruitment of macrophages during pristane-induced inflammatory response in the spleen.

## 4. Discussion

In the present study, chronic inflammation was modeled by injecting pristane into the peritoneal cavity of mice. The regulation of leukocyte recruitment to the spleen during chronic inflammation was mapped utilizing mice deficient in B cells, T cells, or genes known to participate in the inflammatory response. Recruitment of macrophages to spleen required splenic T cells during pristane-induced inflammation. B cells were important not only for macrophage recruitment but also for dendritic cell and neutrophil recruitment. Additionally, IL-6 promoted the recruitment of macrophages to the spleen. Moreover, LT*α* enhanced the recruitment of macrophages and neutrophils. However, TNF*α* deficiency slowed the recruitment process of neutrophils and macrophages to the spleen and TNFR1/2 promoted the recruitment of macrophages and neutrophils in a similar manner to B cells.

As a major cell type in spleen, B cells play a crucial role in regulating the immune response. B cells can regulate experimental autoimmune encephalomyelitis (EAE) via the production of interleukin-10 (IL-10) [20]. Induction of B cell-derived IL-10 provides protection against EAE [21]. Furthermore, IL-10 derived from B cells restrains the development of ulcerative colitis by suppressing T helper (T_H_) 2-type immune pathology and reduces the production of T_H_2 cell-mediated IL-4, which can trigger the disease [22, 23]. Although B cells may not act in the same way in different diseases when regulating the immune system, B cell deficiency may provide protection against chronic inflammation, or perhaps the absence of B cells can alter antigen presentation by dendritic cells and macrophages to B and T cells, in turn perturbing the feedback regulation between lymphocytes, dendritic cells, and macrophages. We previously reported that the absence of B cells almost fully inhibited the recruitment of dendritic cells, neutrophils, and macrophages to the peritoneal cavity during pristane-induced chronic inflammation [10]. A similar regulatory pattern was observed in the spleen for the present study, suggesting that B cells also are crucial in mounting a noninfectious inflammatory response. In contrast to B cells, T cells play a limited role in pristane-induced inflammation; only macrophage recruitment required T cells. Pristane-induced arthritis has been proposed to be CD4^+^ T cell dependent [24].

Differential roles of B cells and T cells in regulating pristane-induced inflammation suggested that cytokines may play differential roles in regulating pristane-induced noninfectious chronic inflammation. Our results indicated that TNF*α* helped quickly mount the inflammatory response to pristane treatment. Lack of TNF*α* receptors resulted in a limited response to pristane treatment in the spleen and in the peritoneal cavity. The use of an anti-TNF*α* antibody or recombinant soluble TNF*α* receptors to inactivate TNF*α* ameliorated inflammation within mice with collagen-induced arthritis [25]. TNF*α* AU-rich elements (AREs) are targets for p38 and JNK kinase signaling, and deletion of TNF*α* AREs leads to the overexpression of TNF*α*, further exacerbating chronic inflammatory arthritis and inflammatory bowel disease [26]. Unlike TNF*α*, dendritic cell-derived LT*α* modulates the production of IgA and cellularity of secondary lymphoid tissue, in turn influencing dendritic cell homeostasis [15, 27, 28]. The absence of LT*α* signaling blocks IgE synthesis and disrupts the balance in mouse CD4^+^ T_H_ cell subsets, as well as in the spleen and lungs of mice lacking LT*α* express higher levels of T_H_1-type cytokines [29]. Herein, we demonstrated that a lack of LT*α* blocks the accumulation of macrophages and neutrophils in the spleen and in the peritoneal cavity during pristane-induced inflammation. LT*α* and TNF*α* have been shown to play independent roles in the evolution of the granulomatous response in experimental leprosy because of their distinctive roles in the recruitment of lymphocytes. Unlike TNF*α* and LT*α*, IL-6 is only required for macrophage recruitment to the spleen in response to pristane treatment. Therefore, chemokine expression may be regulated differentially in various pathologies associated with the expression of different cytokines. This would need to be clarified in future studies.

Furthermore, cytokines play differential roles in directing leukocyte accumulation to the spleen or to the peritoneal cavity during pristane-induced noninfectious inflammation. IL-6 promotes the recruitment of both neutrophils and macrophages to the peritoneal cavity; but in the spleen, it directs the recruitment of macrophages only. TNF*α* delays the recruitment of neutrophils and macrophages to the spleen; however, it promotes the clearance of neutrophils and blocks the recruitment of macrophages in the peritoneal cavity. Unlike to IL-6 and TNF*α*, a lack of TNF receptors inhibits the recruitment of both neutrophils and macrophages in the spleen, but only neutrophils in peritoneal cavity. It seems that the migration of leukocytes is tightly controlled during noninfectious inflammation.

In conclusion, during pristane-induced non-infectious inflammation, the recruitment of dendritic cells to the spleen requires TNF*α* receptors and the presence of B cells. For neutrophil recruitment, TNF*α* and LT*α* are required, in addition to TNF*α* receptors and B cells. However, macrophage recruitment to the spleen is controlled by more factors, including IL-6 and T cells, as well as the other regulators for neutrophil recruitment. This study may provide insight into the development of chronic inflammatory diseases associated with noninfectious stimuli.
